# Treatment-seeking behaviour among infertile tribal women of Palghar district in India

**DOI:** 10.3389/frph.2023.1115643

**Published:** 2023-10-25

**Authors:** Arvind Santu Jadhav, Dipti Govil

**Affiliations:** International Institute for Population Sciences (IIPS), Mumbai, India

**Keywords:** tribal infertility, traditional healer, treatment rituals, health care, tribal community

## Abstract

**Background:**

The tribal culture views health care practices differently from the mainstream. Infertile tribal women practice treatment-seeking behaviour that reflects the community's cultural access to and availability of treatment as well as customs to meet their health care needs. In the environment of rising awareness of infertility and numerous treatment options, this study aims to highlight the infertility treatment-seeking behaviour of tribal communities.

**Data and methods:**

The study employed a mix methods approach to collect data from 153 tribal couples suffering with infertility (interview schedule) and the community (in-depth interviews—14 and focus group discussions—12). The data was analyzed using Stata 13.0, and NVivo 10. Results of the quantitative data were triangulated with qualitative data for writing.

**Results:**

Many treatment options were available for infertility in the community. Generally, traditional healers (98.7%) were preferred over modern healthcare practitioners (35%). Community members usually guided infertile couples for choosing providers. Treatment practices were very different among primary infertile women than sub-fertile. Women frequently discontinued treatment and switched between providers because of unaffordability, poor connectivity, distance, travel time, time constraints, and non-supportive circumstances. The couples were advised to consume herbs, and eat or avoid certain food items, were given massage, burns on the abdomen *(dagani)*, removed black blood (*phasani*) and other various rituals to appease spirits, get rid of ghosts while offering animal sacrifice, and conducting *shidwa ritual.* The mean expenditure on treatment was INR 18,374. If treatment did not yield any result, couple resorted to add another wife, divorce, accept childlessness and foster a child.

**Conclusion:**

Local authorities should strive to work towards the socio-economic development of the tribal communities and provide good healthcare services at their doorstep. The infertility problem needs to be understood in the context of poverty, tribal beliefs, and unequal access to healthcare resources.

## Introduction

Infertility, or the inability to become pregnant after unprotected cohabitation for 12 months (1), is a universal public health challenge that transcends cultures and affects millions worldwide (2). Estimates suggests that more than 48 million couples and 186 million individuals live with infertility across globe (3) with an overall prevalence of 9% (4). Infertility can be divided into primary or secondary; where primary infertility includes inability to conceive, while secondary infertility refers to failure to conceive following a previous pregnancy. Globally, the primary and secondary infertility rate of women aged 20–44 is 1.9% and 10.5% respectively (3). In developed countries, the prevalence of infertility ranges from 3.5% to 16.7%, and from 6.9% to 9.3% in the developing world (5). In India, the infertility prevalence stands at 8.9% (6, 7). Factors like sexual history, lifestyle (stress, obesity) and cultural background of the people and postponing parenthood increase the prevalence of infertility (8, 9).

Infertility are attributable to both men and women; their experiences differ significantly. Regardless of the medical causes of infertility, men are rarely considered infertile, and the obligation to negotiate with infertility falls on woman (10). She endures majority of the fertility testing and treatment (11). Studies report that infertility has several social, economic, and psychological implications (12, 13) particular in the societies, including India, where it carries significant social stigma (14).

The social consequences can be grave as parenthood is desirable in many societies; children are expected to provide care and maintain their parents, while being part of the social support system (6). They also carry family's name and inheritance and perform the parents' last rites (15). Owing to the importance of children, infertility touches all aspects of a childless couple's life, including their status, respect, and authority (6). Many childless couples, especially women, experience violence, divorce, social stigma, emotional stress, depression, anxiety, low self-esteem, a sense of failure, and exclusion (16). They are ostracized and lose their status in the family and society.

Due to familial and societal pressures, many infertile women or couples resort to different treatment options and practices. The choice of treatment is strongly influenced by their awareness and knowledge of biomedical, faith-based, and traditional options (17). The attitude towards treatment seeking is also influenced by social structure, socialisation, and experience with infertility and childbearing (18). Forced by social pressures, childless couples often undergo repeated “trial and error” treatments (19).

As advanced investigations are fundamental for the treatment of infertility, the availability of allopathic treatment services is essential (4). Across the developing world, there exist wide disparities in the quality, availability, and delivery of infertility services; hence, most infertile people have poor access to effective treatment (20). In India, modern infertility treatment including ART is available in both public and private sectors. The public sector offers this treatment through tertiary hospitals/medical colleges and a few basic investigations at lower levels (21, 22). However, the quality of treatment in the public health sector, the only treatment affordable for rural and poor Indians, is relatively poor. Treatment facilities outside the government health systems are prohibitively expensive because of which they primarily cater to the educated elite who can afford such treatment. Literature suggests that ART services are not accessible to the majority of infertile couples due to the high cost of treatments in addition to cultural, religious and legal barriers (23). The cost also forces several couples to stop the treatment or shift to other treatment options (20). A nationally-representative Indian study, using data from the Demographic and Health Survey, revealed that in rural India, the treatment of infertile women begins with allopathy and ends with traditional healers (24).

Couples with primary infertility are usually more interested in treatment than those with secondary infertility (25), irrespective of who the infertile person is among the couple. Though there is an awareness among couples of the possibility of infertility when pregnancy is delayed (26), women are less knowledgeable about the causes of infertility and the options for treatment (27) However, it is usually the woman who initiates the first contact with a practitioner (28). Strong cultural beliefs and healthcare-related factors are crucial in deciding the treatment mode. Conventional methods and religious practices include consultations with unqualified herbal and spiritual (traditional) healers and diviners and visits to religious places like temples, churches, or mosques (12). Infertile couples may also observe tantric rites, wear charms, visit astrologers, and follow prescribed rituals (29) as community strongly believe that supernatural powers cause infertility.

In resource-poor rural communities, there is an overwhelming dependence on traditional healers, irrespective of caste and class (30). Low education, insufficient knowledge and awareness (31), residence in remote areas, dependence on agriculture, and different belief systems make the tribal population distinct from the other sections of the population. According to a study conducted in Madhya Pradesh, tribal communities exhibited a higher prevalence of infertility compared to other populations, which may result in distinct patterns of coping or treatment within these communities (32). In the background of rising awareness of infertility and numerous treatment options, few Indian studies have explored the infertility treatment-seeking behaviour of tribal communities. The study aims to bridge this gap by examining the treatment-seeking behaviour of infertile tribal couples and understanding the hardships faced by them during treatment.

The study's theoretical framework is anchored on a socio-cultural perspective that recognizes the influence of cultural beliefs, norms, and practices on the experiences of infertility treatment in tribal communities. The study aims to generate a comprehensive understanding of infertility treatment and its impact on tribal couples’ lives, as well as to identify the cultural factors that shape their practivces and attitudes towards infertility and its treatment. The mixed-methods approach employed in this study is well-suited to capture the complexities of the cultural and social dynamics underlying the experiences of infertility treatment among tribal communities. The findings of this study can inform the development of culturally sensitive interventions and policies to address infertility-related issues in tribal communities, particularly in resource-poor settings like Jawhar tehsil.

### Data and methods

A primary study covering 153 tribal couples, who had ever experienced infertility, was conducted in Jawhar tehsil of Palghar district, Maharashtra, in 2016–17. Despite being surrounded by the relatively better-developed union territory of Dadra & Nagar Haveli, and the metropolitan areas of Nashik and Mumbai, a large proportion (83%) of the tribal population in Jawhar tehsil (91.6% tribal population) lives below the poverty line (33). Jawhar is located in a hilly zone far from the district headquarter and possesses poor civic and health facilities. The population is scattered across the villages in small hamlets called *padas*. Each *pada* accommodates a population of 60 to 600. A total of six villages in Jawhar tehsil were selected for the survey based on their distance from the district headquarters, the concentration of the population, and the predominance of the Warli tribe. Through a complete mapping-listing, 153 eligible couples (43 primary infertile^1^ and 110 sub-fertile^2^) were identified and interviewed. Data was collected from both emic and etic perspectives. An interview schedule was administered to get information from the infertile couples primarily women. The tools were tested before main survey. The quantitative data was analyzed for descriptive and inferential statistics using Stata 13.0.

In-depth interviews (IDIs) and focus group discussions (FGDs) were held to gather information from the community. A total of 14 IDIs and 12 FGDs were conducted in six villages. From each village, two FGDs (men and women separately) were conducted to get diverse views, perceptions, opinions, and practices related to infertility. The respondents of FGD were older adults (married and with children), and for IDIs were community representatives like healthcare providers, the village head, and traditional healers (Bhagat or Bhagtin). The number of participant in the FGD ranged from 6 to 10. IDIs and FGDs were conducted in local language with the help of pre-tested and translated (Marathi) guideline. The subjects for qualitative interviews were identified during mapping and listing operation along with study sample and were requested to participate in the discussion during main survey. Researcher with the help of a female investigator facilitated the discussion in FGDs in gender balanced environment. Female investigator also helped in handling any sensitive information. The discussion was audio recorded along with the field notes and observations with due permission. FGDs were conducted in the common places within the village/community such as community hall, pre-primary education center or at any other mutually decided and comfortable places within the community.

FGDs and IDIs were transcribed verbatim and field notes were incorporated to get the correct scenario. The data was thematically analyzed using NVivo 10. Both inductive and deductive coding techniques were used to analyse the qualitative data. Based on the guidelines and objectives of the study, the broad themes were generated. The themes or coding structure were refined and strengthened after reading the transcripts several times. These themes and sub-themes were then applied to all transcripts. Later, the entitle coding structure was translated in NVivo. Primarily, the broad themes included causes of infertility, type of treatment options for infertility in the community (traditional and modern), features of different treatment practices, rituals followed in different treatment options, advises during treatment, advices for couple who face infertility problem, community treatment to couple, coping strategies, and precaution to avoid infertility.

The rights of the participants were ensured and safeguarded with standard practices in ethical research. The procedures and tools used in the study were approved by the Institute's “Students Research Ethical Committee” before their implementation in the field. Informed consent of the participants was obtained before the study's commencement; privacy and confidentiality of information were ensured throughout. Furthermore, the principles of reciprocity and reflexivity were adhered to during the fieldwork. A female investigator was recruited from each village to support the data collection process. Her presence was essential during the interviews to administer sensitive questions and make infertile women feel comfortable.

## Results

**Profile of the respondents:** Mean age of the respondents in the sample was 29.8 years. Average age gap between husband and wife was 3.4 years. Fifty-four percent respondents were literate and 68.6% had exposure to mass media (television, newspapers, radio). Seventy-one percent respondents belonged to Warli tribe. On an average respondents were married for 12.5 (±7.5) years. Nearly 65% respondents were undernourished and only 2% had high BMI.

**Treatment options for infertility in the study area:** Several traditional and medical treatment options are available for couples with infertility in the study area. Infertile couples first access the options (primarily traditional treatment), which are available within their own village. Once traditional treatment does not yield any result (pregnancy), one of the spouses goes out of the village to seek alternative healthcare services (traditional or modern). Treatment is frequently switched between wife and husband (although men are rarely consulted or undergo any long-term treatment) and traditional and modern. The community members usually guide such couples while choosing treatment providers. The treatment options for infertile couples in the tehsil are described in Table 1.

**Table 1:** Treatment options and procedures for infertility care in the tribal communities of Palghar, Maharashtra.

Treatment providers	Description
**1. Dai (Soyrin)**	A traditional birth attendant is locally known as *dai* or *soyrin*. She* *is usually a senior member of the community, who guides people on various health issues and treats ailments apart from handling childbirths. She supplies herbal medicines or gives massages and advice to infertile couples.
**2. Faith healers**	** **
***Bhagat/Bhagtin*** 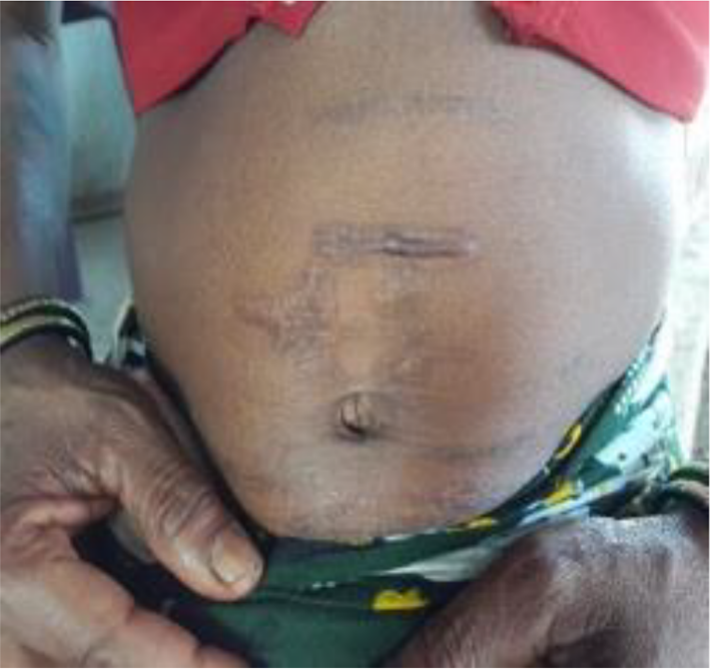 Burn marks on the abdomen of an infertile woman after*** dagni ***treatment by a ***bhagat***	*Bhagat, *the most prominent traditional healer in the community*, *provides healthcare services and consultation on various other matters. The community is familiar with his schedule, treatment procedures, and cost and hence prefers to visit him. *Bhagat's* treatment is considered superior to other options. Most infertility-related issues are resolved here. He conducts rituals at the couple's house, mosque, or temple. His usual approach is to diagnose the “ghost tumour” *(Bhutgat*) in the abdomen of an infertile woman, which is believed to be the cause of menstrual problems and infertility. Once the “ghost tumour” is identified, the treatment includes massage, application or consumption of herbs (roots, leaves, barks, and flowers), and use of amulets and other talismans (to be worn; hung on or nailed to the walls of the couple's home; or buried) to ward off evil spirits or nullify the negative influences of supernatural powers. *Dagni*, a specific ritual to dissolve *Bhutgath,* includes pouring drops of mild acid on the abdomen or burning abdominal skin with a red-hot nail or iron stick. With this treatment, a woman's menstrual cycle returns to normal and her chances of becoming pregnant improve. Advice and instructions to avoid or consume certain food items and practice things in a certain way are also given. As many visits as suggested by the healer should be made. Whether the couple conceives or not, the rituals (payment of treatment) must be executed within a year, or the curse of bhagat will aggravate the infertility problem. During every visit, patient needs to offer INR 50/100 to the deity in the *bhagat's *house. The entire treatment costs around INR 2,000–10,000. 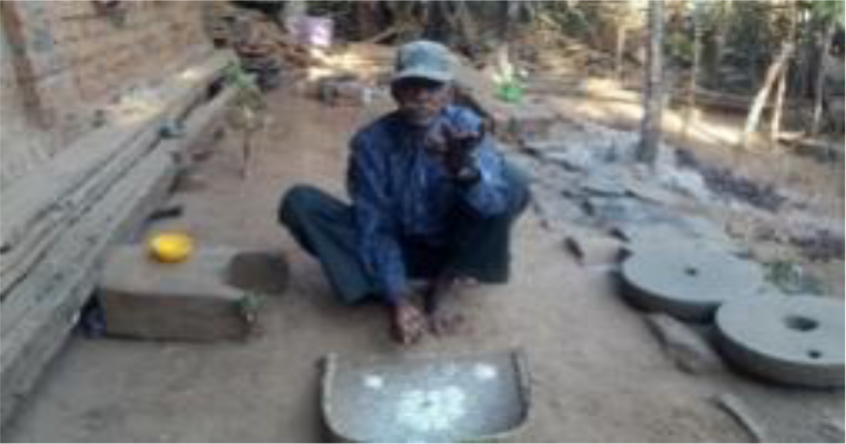 ***A Bhagat ***performing rituals for infertility treatment. 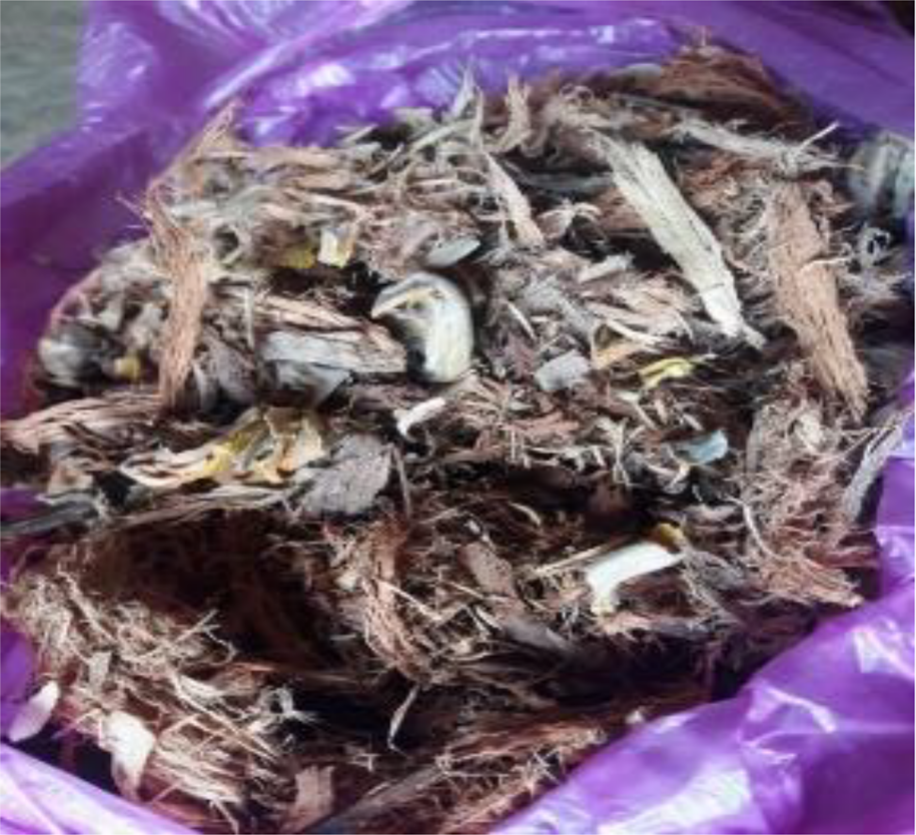 ***Herbal medicines ***dispensed by a*** Bhagat (for application on the body and consumption after boiling)***
*Nandiwala* 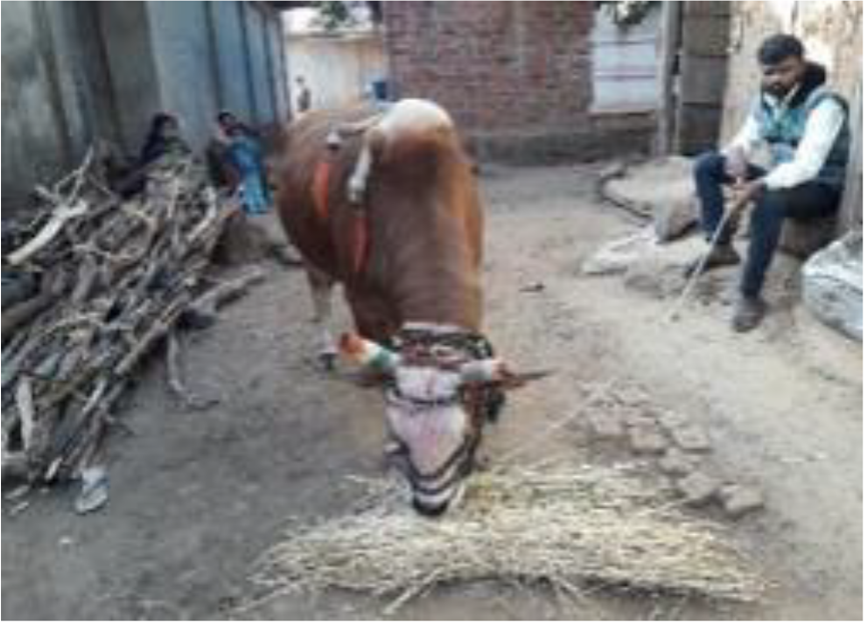	Apart from the *bhagat,* blessings and medicines can also be sought from a *nandiwala* (a person who carries a deformed bull and roams from village to village). He visits villages and collects offerings and gifts for advice on healthcare and treatment with herbal medicines. The *nandiwala* leads a nomadic life and is ethnic to the state. He plays a drum to announce his visit to the village or house. Women with infertility pray to the bull and receive blessings. The *nandiwala* also gives betel nuts vomited by the bull to be tied in the corner of a saree (Indian traditional attire) of the infertile woman. It is believed to be a symbol of blessings from the *nandi*, i.e., bull. The *nandiwala* receives INR 1,000–2,000 in return.
*Navnathwala* 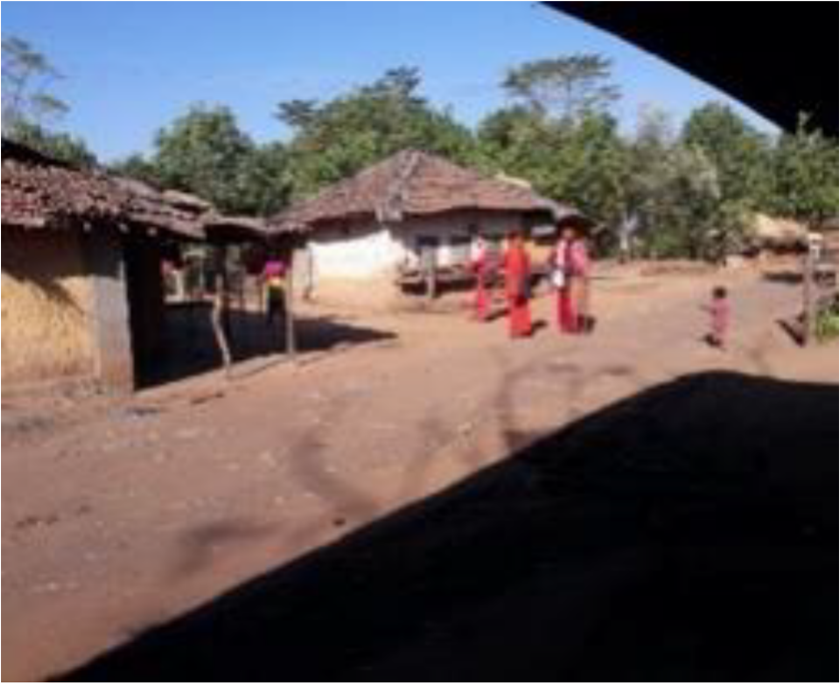	*Navnathwale,* the faith healer, is a devotee of saint/God *Navnath* and hails from the Trimbakeshwar region of Nashik district. He is believed to be vegetarian, non-alcoholic, and follow bachelorhood. He survives on donations received in cash or kind. He treats infertile couples by reading palms, narrating motivational stories, giving advice for fasting, recommending which food items to eat or avoid, and recommending praying regularly to *Navnath*. He, in return, receives INR 51, 101, or 501.
*Ghubadevi* 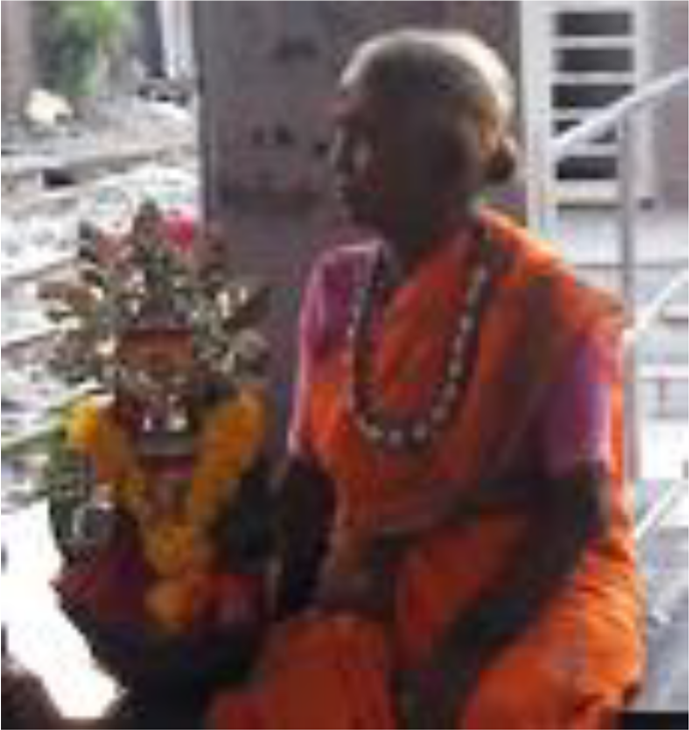	*Ghubadevi* is the name of a goddess. A woman devotee carries the idol of *Ghubadevi*, placed in a bamboo basket, from door to door after the harvesting season. The copper, gold, or silver-coated face of the idol is visible from the basket, and the idol is wrapped with a green cloth, symbolising prosperity. The forehead of the idol is covered with *haldi-kumkum* (turmeric and vermillion). The woman devotee applies this powder on the forehead of an infertile woman as a blessing of the Goddess. In return, she gets food grains like rice, pulses, and cash or coins.
*Hijara* (LGBTQ)	Blessings of the transgender community is sought as a treatment for infertility.
**3. Prayer to God, deities, and religious faith healers** 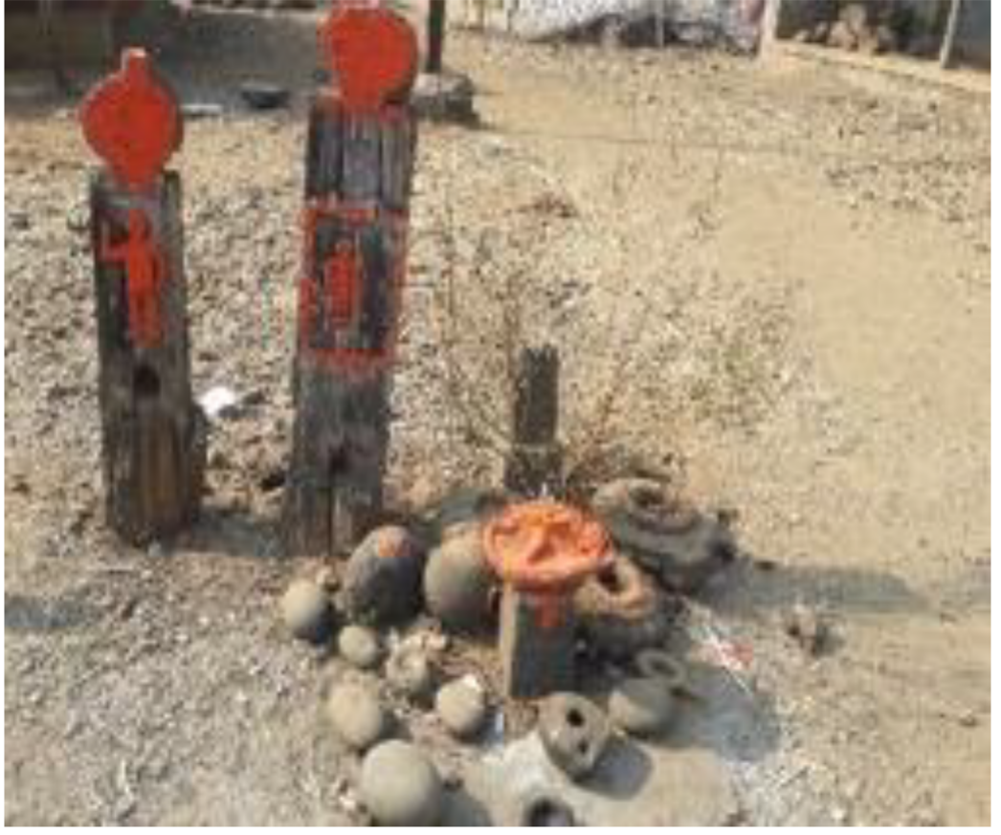	The well-known Indian festival of colour, *Holi, *is believed to cure infertility issues.* *A prayer called* Navas *is organised* *specially* *for newly married couples to resolve their fertility issues and bless them to have healthy children. If a woman conceives after this prayer, the couple or family either offers animal sacrifice or distributes coconuts, jaggery, sweet bread (*pooran poli*), *papad*, and sweet rice to villagers during the next Holi celebration. No one denies or rejects this offering. The community also mentioned about the prayers of other deities such as *Shri Krishan*, *Lord Ganesh, Hirva Dev, *Goddess M*ahalaxmi, Saptsrungi, and Jivdani.* Apart from this, the belief in faith healing is underscored by the villagers as they worship various saints or *pirs,* including *Narendra Maharaj, Samarth Ramdas Swami (Hindu), Dargahwala Baba (Muslim), and Yeshuwala Sevak (Christian)*. The rites and rituals include fasting, worship, and chanting prayers, among other things.
**4. Healthcare providers from the allopathic stream**	Allopathic treatment (modern medicine) is sought mainly from public sector health facilities. Private facilities are very rarely accessed. The choice is influenced by various factors like proximity to the village, familiarity and comfort level, and cost. In the public sector, the first choice is primary healthcare centres, where the couples receive some guidance or medicines.

**Treatment-seeking behaviour for infertility:** Except two, all infertile respondents sought treatment from a faith/traditional healer, locally known as *bhagat* (Table 2). A handful respondents (44.2% of primary infertile and 30.9% of sub-fertile) also sought treatment from the allopathic stream. However, the majority did not continue with it. On an average, couples consulted 0.4 allopathic practitioners and 4.4 faith healers. Couples with primary infertility visited more faith healers (5.8) than sub-fertile women (3.8). Median number of consultations were 22 over 3.5 years. About half of the respondents consulted a treatment provider for infertility after 24 to 36 months of marriage/last pregnancy or birth. Most sought treatment within their taluka because of less travel time. The usual travel time to reach a provider was still as high as one to two hours and they walked the distance for treatment.

**Table 2:** Treatment-seeking behaviour among couples who experienced, or were experiencing infertility, Palghar.

Background characteristics	Primary Infertile	Sub-fertile	Total
*n*	43	110	153
Type of providers consulted^a^			
Health provider from allopathic stream	44.2	30.9	34.6
Traditional providers (primarily Bhagat)	100.0	98.2	98.7
***n***	**43**	**108**	**151**
Mean number of providers visited	6.9	4.3	5.0
Mean number of allopathic healthcare providers visited (*n *= 53)	0.4	0.3	0.4
Mean number of faith healers visited (*n *= 151)	5.8	3.8	4.4
Median number of consultations	32.0	19.5	22.0
Average duration of treatment (in years)	2.9	3.8	3.5
Average gap between marriage/last pregnancy or birth and initiation of treatment (in months)	30.1	28.7	29.1
Location of treatment provider—within tehsil	51.2	69.4	64.2
Time to reach treatment provider (1–2 h %)	79.1	75.0	76.2
Mode of transportation (walk) (%)	93.0	95.4	94.7

^a^ Multiple Responses.

The bold values refer to sample size (small n). The percentages in the following rows are from that (sub) sample size (*n*), instead of the whole sample (*N*).

**Figure 1:**
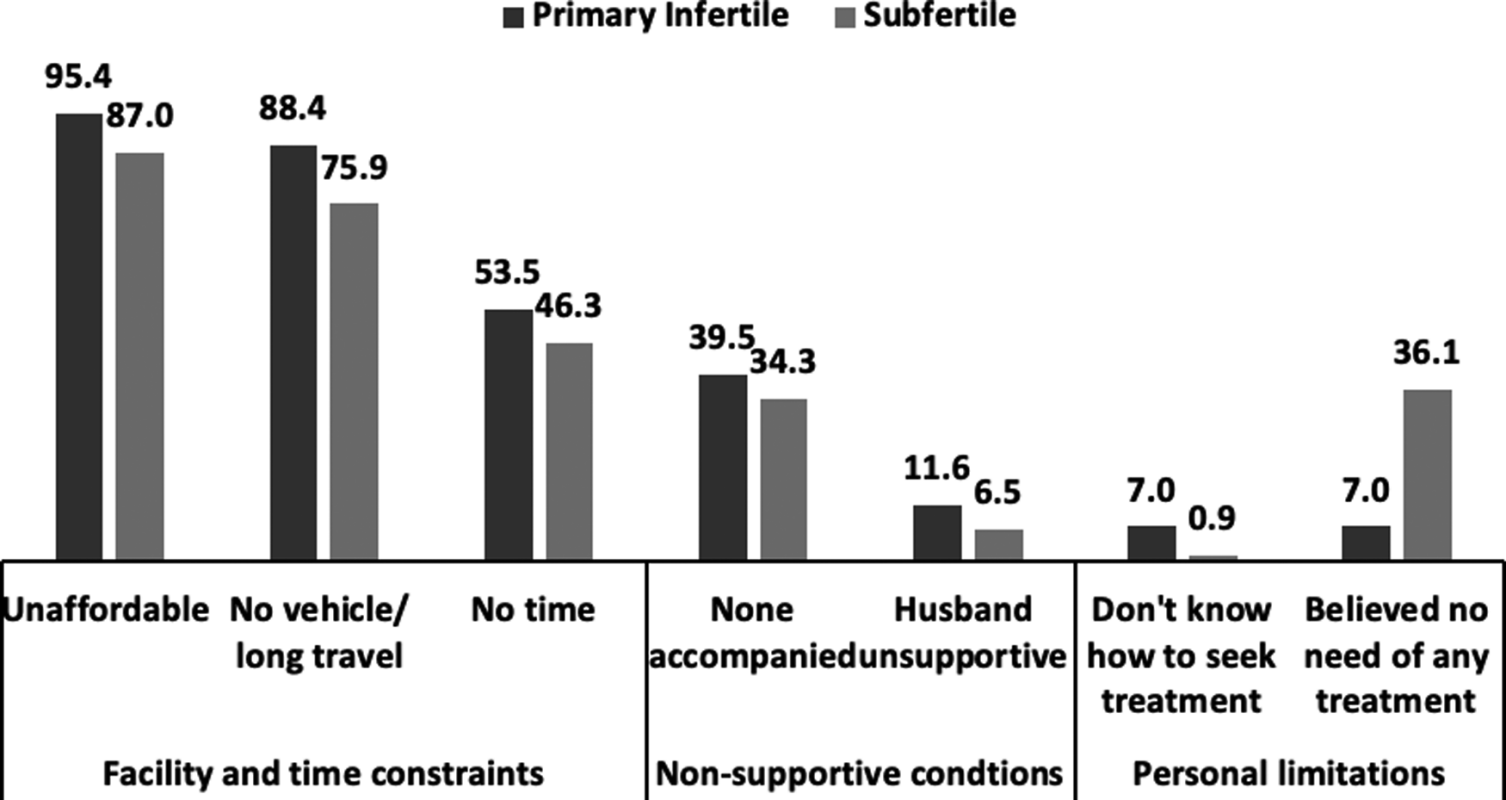
Reasons for changing providers or discontinuing treatment (any) for infertility, Palghar (*n *= 151).

The primary reasons for discontinuing a treatment and frequently switching between health providers included unaffordability, poor connectivity, long distance or travel time, time constraints, and non-supportive circumstances (have to travel alone or have an unsupportive husband) (Figure 1). Even if a couple was referred to a public healthcare facility in a nearby district, they would not have sufficient money to travel to the facility or start treatment. There was also a fear to travel to new places as most tribal respondents never travelled outside their village or tehsil. Additionally, the couples hesitated in sharing their problems with unknown people (formal healthcare providers). Community too generally discouraged couples from leaving the village, usually with the stories of theft, cheating, and fraud. Despite the barriers, a few couples consulted a doctor; however, they could not comply with the treatment protocols and procedures because of their high expectation of getting cured with a single pill or in one visit. After realising that the expectation could not be met, the couple withdrew from the treatment.

Those who visited government facilities were unhappy with the quality of care received. Many respondents mentioned (off the record) that they received harsh treatment from healthcare professionals. Patients from a particular section of the society (primarily rich/higher community) received preferential treatment, while the others were ignored. They also complained of paramedical staff being drunk on duty. Some respondents said they stopped the treatment because they did not like being scolded by the doctors over the *dagani* (burn marks on the abdomen). Based on the opinion of community members, the differences in treatments received from faith healers and doctors can be categorised as (a) accessibility and affordability, (b) convenience, (c) trust and respect between patient and service provider, (d) features of the treatment, and (e) side effects of the treatment.

“*The staff at the government hospital do not give [us] adequate attention and care and spend no time to discuss the treatment options. At private facilities, they just inject medicines and take money from us* *for no reason.”* (translated)*—reported by* a 29-year-old primary infertile woman, who had undergone treatment for seven years.

Another primary infertile woman aged 23 years, who had been having treatment for four years, added: “*When we visit the local PHC (Public Health Centre), the doctor is often absent. The doctor at a nearby PHC resides far away. In case of an emergency, especially at night, he does not respond to calls. In fact, no one in the PHC attends to us. Therefore, we need to travel to the sub-district by bike and private care, 15 km away from home, for any emergency. Considering the state of affairs at the government facilities and the distance to the private health centre, we decided to visit the bhagat only.”* (translated).

**Figure 2:**
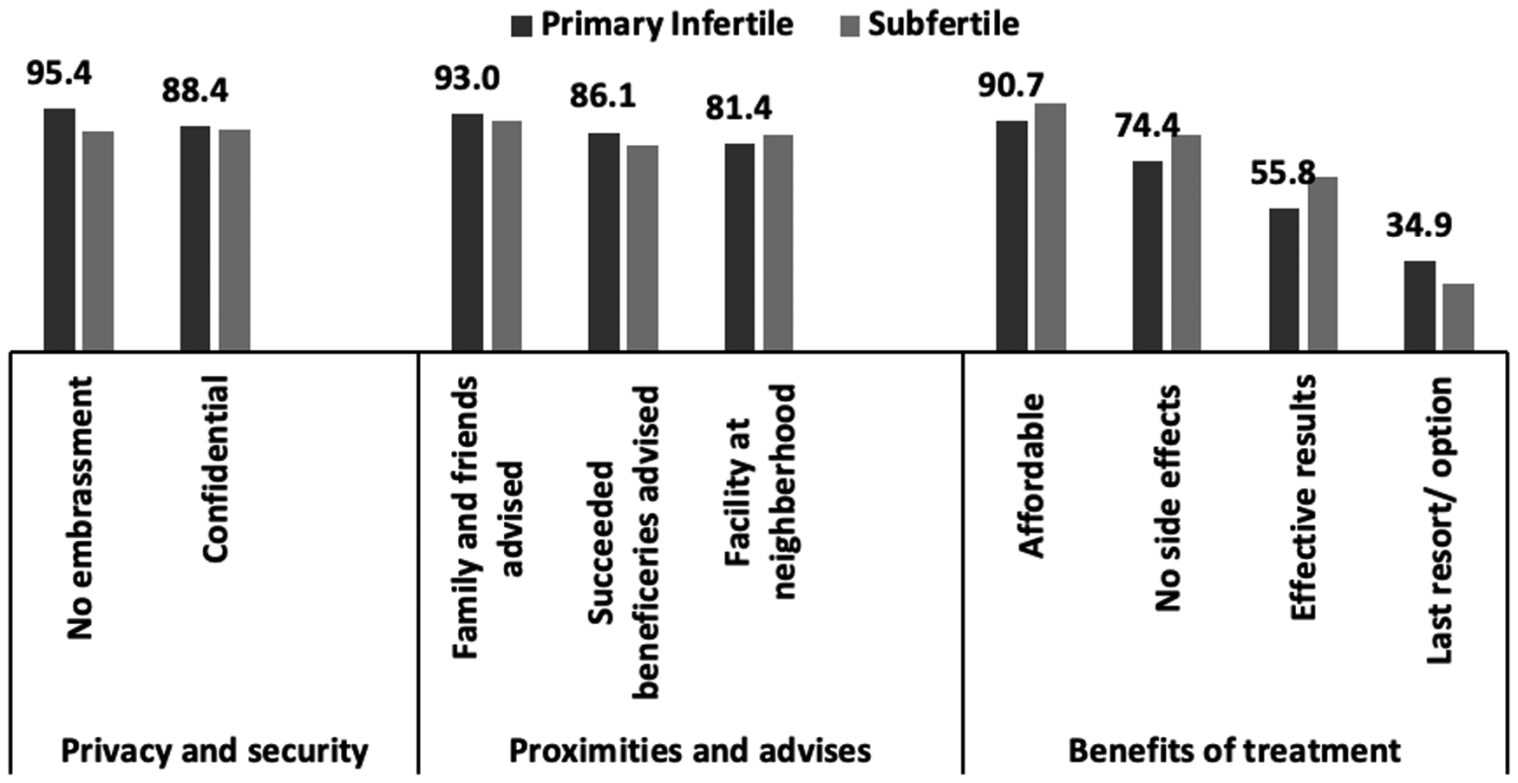
Reasons for choosing faith healer to treat infertility Palghar (*n *= 151).

Generally, faith healers were preferred over practitioners of modern medicine because of the perceptions that the causes of infertility lie in supernatural influences and witchcraft, the faith healers take better care of patients, and their treatment is effective. A 32-year-old illiterate woman with two children said, “*No government hospital (sub-district level healthcare centre) gives [us] as much attention as bhagat [does]”* (Translated). Other reasons for preferring these healers were privacy, proximity to home, recommendation of family and friends, affordability of treatment, and absence of side effects (Figure 2).

**Traditional treatment, rituals, and restrictions:** Of the women who visited faith healers, 91.4% followed treatment advice. The couples *were* advised to consume certain herbs (96%) and eat or avoid certain food items (61.6%). A *bhutgath* was identified in 86.1% and massage was given to 88.1% respondents. The women said they were comfortable with the faith healers touching them. Fifty-three percent received burns on the abdomen (*dagani)* and had black blood (*phasani*) removed. The faith healers performed various rituals to appease spirits, get rid of ghosts and offered animal sacrifice (Table 3). The rituals are carried out in the following order: witchcraft, prayer, offerings, massages, advice, herbal medicines, and amulets.

**Table 3:** Type of rituals and activities in infertility treatment, Palghar.

Types of rituals/activities^a^	Primary Infertile	Sub-fertile	Total
*n*	43	108	151
Followed treatment advice	90.7	91.7	91.4
***n***	***39***	***99***	***138***
Type of rituals			
Massage of abdomen	90.7	87.0	88.1
Eating or avoiding certain foods	67.4	59.3	61.6
Assessed for tumour (*bhutgath*)	88.4	85.2	86.1
Applications			
Ash on the forehead	88.4	92.6	91.4
Herbs and roots	76.7	76.9	76.8
Hot object on the abdomen *(dagani)*	55.8	51.9	53.0
Oral			
Roots/Herbs	100.0	94.4	96.0
Drinking holy water	58.1	38.9	44.4
Consuming herbs/roots/leaves in alcohol	39.5	51.9	48.3
Appeasement of spirits or exorcising ghosts			
Performing rituals	86.1	88.0	87.4
Sacrifice of animals	67.4	78.7	75.5
Praying to family Deity	93.0	87.0	88.7
Burying certain objects or hanging them at home	48.8	42.6	44.4
Dropping edibles at crossroads	46.5	48.2	47.7

^a^ Multiple Responses.

The bold values refer to sample size (small n). The percentages in the following rows are from that (sub) sample size (*n*), instead of the whole sample (*N*).

At last, the final ritual named *shidwa*^3^ is performed. The *sidh* (Bauhinia Racemose) tree is the preferred site for performing the *shidwa (*Table 4*).* This ritual can also be performed at the infertile couple's or faith healer's home, or in forest or farm. About 13% of the respondents had *the shidwa* ritual on full-moon (*Poornima*) or no-moon (*Amavasya*) nights. The Diwali festival is the most popular time to conduct this ritual (44.2%) because it comes after harvesting season and people are relatively free during that time. However, the ritual should be performed as early as possible because delay is not appreciated. Breaking coconuts and slaughtering or releasing a cock/goat/chicken are the main offerings during *shidwa*.

**Table 4:** Features of the shidwa ritual, Palghar (*n *= 138).

Characteristics	Primary Infertile	Sub-fertile	Total
*n*	39	99	138
Place for conducting Shidwa ritual			
Under *Sidh* Tree (Bauhinia racemosa)	59	73.7	69.6
Near the riverbank	5.1	15.2	12.3
Other places	35.9	11.1	18.1
Time for Shidwa ritual^a^			
Morning	20.5	18.2	18.8
Noon	43.6	49.5	47.8
Evening/Night	35.9	32.3	33.3
Full/no moon night	16.3	12.0	13.3
Season for ritual^a^			
Holi	33.3	24.2	26.8
Navratra/Dasara festival	10.3	8.1	8.7
Diwali festival	20.5	53.5	44.2
Non-festive time	59.0	16.2	28.3
Person for on whom the ritual is conducted^a^			
Wife	100	100	100
Husband	51.3	91.9	80.4
Child	--	85.9	61.6
**Number of villagers attending the ritual (median)**	5	15	12
Sacrifices offered^a^			
Coconut	84.6	98.0	94.2
Cock (slaughtered or released)	76.9	88.9	85.5
Chicken (slaughtered or released)	53.9	70.7	65.9
Cock or chicken buried	15.4	64.7	50.7
Slaughtering of goat	56.4	88.9	79.7
Burying an earthen pot	7.7	68.7	51.5
Puja material dropped in flowing water	7.7	32.3	25.4
Things used for the rituals^a^			
Cash or coins	92.3	97.0	95.7
Coconut	87.2	100.0	96.4
Rice or grain flour	74.4	87.9	84.1
Alcohol (made with jaggery or *Mohua* flower)	69.2	91.9	85.5
Clothes	53.9	82.8	74.6

^a^ Multiple Responses.

The bold values refer to sample size (small n). The percentages in the following rows are from that (sub) sample size (*n*), instead of the whole sample (*N*).

The treatments administered by the *bhagat* are not without side effects. Some women reported nausea or loss of consciousness after consuming the herbs. Others reported swelling of the eyes, restlessness, and severe pain in the abdomen. A woman also feels restless or gets severe pain after *dagni*. The frequent demands for money and alcohol from the *bhagat* also create difficulties for many infertile couples. The traditional healer advises infertile couples to observe certain restrictions during the course of treatment (Table 5). These restrictions are mostly associated with women's behaviour in public places and at home as also with grooming, clothing, and food.

**Table 5:** Advice given by faith healers to infertile women, Palghar, 2016–17 (*n *= 151).

Activities to be avoided^a^	Primary Infertile	Sub-fertile	Total
*n*	43	108	151
Avoid following activities in public places			
Washing clothes at the river or well	79.1	66.7	70.2
Bathing in the river or well	79.1	68.5	71.5
Seeing a dead body or attend funerals	79.1	68.5	71.5
Use a stone or tile that is used by others for washing clothes	76.7	61.1	65.6
Helping others to lift water-pots	72.1	58.3	62.3
Talking to others while fetching water	72.1	63.0	65.6
Avoid doing these at home			
Drying clothes outside the home	79.1	63.9	68.2
Failing to give alms or food to beggars or visitors at the doorstep	48.8	50.0	49.7
Oiling hair after a bath	44.2	36.1	38.4
Restrictions related to food			
Only dal and rice must be eaten	53.5	46.3	48.3
Non-vegetarian	80.6	75.3	75.7
Other food items (Coconut/Turmeric powder/Salt/Oil)	68.8	61.7	63.7
Avoiding the following			
Food cooked by menstruating woman	83.7	73.2	76.2
Food cooked by others	81.4	69.4	72.9
Failing to fast regularly	23.3	21.3	21.9

^a^ Multiple Responses.

The bold values refer to sample size (small n). The percentages in the following rows are from that (sub) sample size (*n*), instead of the whole sample (*N*).

The community members were vocal in their opinion that the women should follow the *bhagat's* advice strictly and avoid quarrels with other women. It was believed that the restrictions were good for developing harmony in the community. The analysis indicates that infertile women are often advised to avoid non-vegetarian food items like eggs, chicken, meat, red blood fish, *Singada,* and dry fish like *Bombil*. Specifically, foods or spices of white and yellow colours, such as coconut, turmeric, salt, and oil, are to be avoided. These restrictions are to be followed even after the completion of the infertility treatment, during pregnancy, and until the infant is six months old and starts consuming supplementary food.

Other treatment characteristics: Of those seeking treatment from the formal health care system, 74.6% infertile women were prescribed medicines for menstrual problems, and 58.2% got laboratory tests (Table 6). Injections were given to more than one-third of infertile women; 16.4% were advised surgery. Nearly one-fifth were told to take an ultrasonography examination. Only 20.9% of the husbands received any treatment for infertility (39.5% primary infertile, 13.6% sub-fertile). Women from a higher standard of living had two times higher chances to avail treatment from an allopathic stream, while other background characteristics like type of infertility, age, literacy, type of tribe, and body mass index were not statistically (table not provided).

**Table 6:** Other treatment characteristics, Palghar.

	Primary Infertile	Sub-fertile	Total
*n*	19	33	52
Allopathic treatment characteristics (women)^a^			
Laboratory test	100.0	52.8	58.2
Tablets for menstruation problems	94.7	63.9	74.6
Other medicines	84.2	100.0	100.0
Injection	31.6	38.9	36.4
Surgery	10.5	21.2	16.4
Ultrasonography	15.8	24.2	21.2
***n***	***43***	***108***	***151***
Treatment seeking among husbands			
Allopathic practitioner	25.6	6.5	11.9
Traditional healer	23.3	10.2	13.9
***n***	***43***	***108***	***151***
Infertility treatment cost			
Mean treatment cost (INR)	18,689	18,530	18,374
Treatment cost in kind—grains, clothes, and alcohol (%)	60.5	61.1	60.9
Sources for treatment expenses^a^			
Self or spouse's earnings	100.0	100.0	100.0
Borrowed money	44.2	50.0	48.3
Sold belongings	23.3	21.3	21.9
Status of the treatment			
Currently taking	55.8	7.4	21.2
Discontinued	44.2	92.6	78.8
***n***	***0***	***100***	***100***
Result of treatment			
Had a son	0.0	45.4	45.4
Had a daughter	0.0	41.7	41.7
Had a miscarriage	0.0	13.0	13.0

^a^ Multiple responses.

The bold values refer to sample size (small *n*). The percentages in the following rows are from that (sub) sample size (*n*), instead of the whole sample (*N*).

Cost of treatment and results: The cost of treatment included cost for travel, medication, consultation, hospitalisation, food, ritual, and gifts. The mean expenditure on treatment was INR 18,374. Most respondents met the treatment expenditure from their earnings. About 48.3% borrowed money, and 21.9% *sold* some of their belongings to meet treatment expenses.

**Figure 3:**
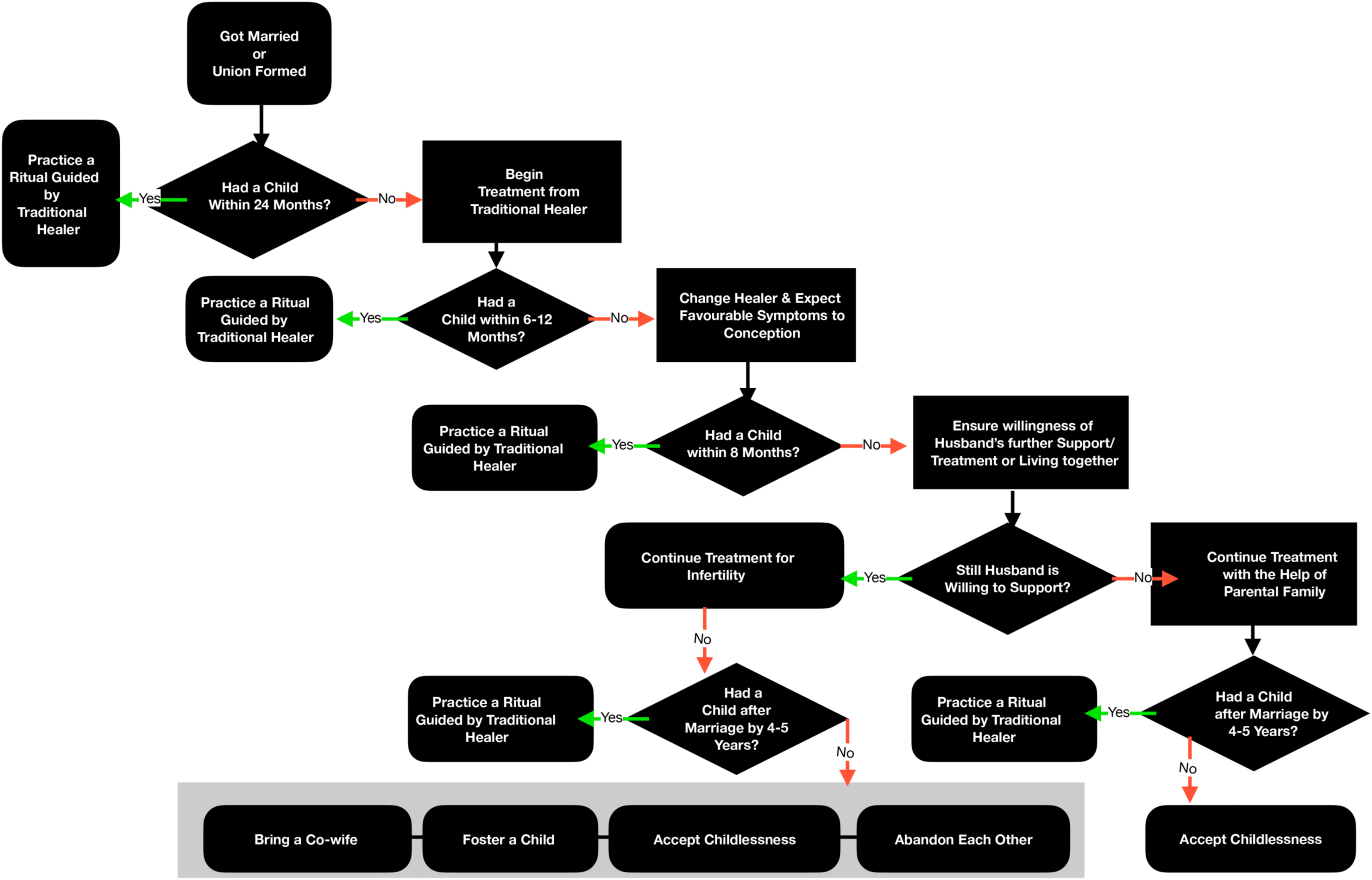
Treatment flow among infertile women: qualitative inputs, Palghar.

At the time of the study, more than half of primary infertile women respondents were still taking treatment. Nearly 44% primary infertile women reported that they had discontinued treatment and were looking for other options. About 92.6% of the sub-fertile women had discontinued the treatment (Table 5) because of successful conception. There is a particular pattern and flow in the treatment-seeking behaviour of infertile couples, as shown in Figure 3. A couple waits for 24 months after marriage or forming a union to conceive. If the woman fails to conceive, the couple first consults a *bhagat* and then visits a medical practitioner for treatment. If the treatment does not lead to a pregnancy, the couple again changes the provider. After 3–4 years of treatment, the further course of action depends on the husband's support. The couple may continue the treatment or decide on one of four actions: the man may bring another wife; the union may end; the couple may come to terms with their childlessness and stop trying; the couple may foster a child.

## Discussion

Children and parenthood are desirable because of several socio-cultural reasons (34). The experience of infertility can be distressing for couples, especially women who suffer from it (13). Appropriate treatment with a timely diagnosis can help couples to have children. However, research suggests that of the total infertile women worldwide, only 56% seek modern medical care (35) that is result oriented. The present paper presents treatment practices related to infertility in the tribal communities of western India.

In the study area, treatment seeking for infertility was universal; except two, all women sought treatment. Multiple treatment options were available, including modern as well as traditional. Treatment options available within the village were the first choice for the infertile couples. This made traditional healers the first contact point to start treatment for infertility. Almost all (98.7%) couples sought treatment from traditional healers within the village or in nearby villages within walking distance. These findings align with the studies which found that infertile women first visit traditional healers and then a hospital (36, 37). Despite the revolution in modern medicine and technology, there has been an overwhelming dependence on traditional or spiritual healers for treatment, irrespective of caste and class, in resource-poor rural communities (12, 38–42).

Treatment seeking from the allopathic stream was limited to one-third of the respondents, much lower than reported by other studies conducted on various other communities (4, 43). It was mainly primary infertile women who sought treatment from the allopathic stream rather than sub-fertile women. This difference may be because of the higher desire of the former to have a child than the latter. The results also show that women with a higher standard of living had higher chances of availing treatment from the allopathic stream. Various studies have established that allopathic treatment seeking is highly associated with higher income groups and general or open castes (20, 24).

People generally chose a treatment provider based on the availability, accessibility, affordability, features and side effects of the treatment and trust and respect between the patient and the treatment provider (20, 40). In the study, infertile couples faced enormous challenges while seeking support from the formal healthcare system. Firstly, the community discouraged them from seeking treatment from the formal system by narrating stories of theft, cheating, bias, unreasonable behaviour of the public sector staff, poor infrastructure, and so on. Secondly, women did not feel comfortable travelling outside the village or taluka because of lack of exposure, limited education, poor connectivity, unaffordability, and side effects of allopathic medicines. Those who somehow managed to go to the formal health system did not believe in long-term care. Such couples were also often chastised by the medical professionals (generally not appreciated) for having availed unsafe treatments from traditional healers, which resulted in burn marks or restrictions on consuming certain food items. There was also a belief that no expert care was available to solve their problems.

The reasons for choosing traditional providers/faith healers included privacy, proximity to treatment, advice from family and friends, and other benefits such as affordability and no side effect. The community also favoured faith healers over allopathy providers because of good care and effective treatment. Literature from Africa revealed that majority women used traditional methods for infertility treatment and demographic characteristics, infertility duration, husbands' relatives' pressure and cheap cost of traditional medical treatment encouraged use of traditional method (44). Couples switched between several healthcare providers either because they could not afford the treatment, could not travel long distances, or because they did not have time to visit a certain provider or had non-supportive conditions in the family or treatment did not yield the result. This is evident from the fact that, on average, women visited 0.4 allopathic and 4.4 traditional providers in 3.5 years, making 22 visits. Primary infertile couples visited more providers (6.9) than sub-fertile couples (4.3). This finding is in line with the findings of other Indian studies (4, 24, 45). Abandonment of a treatment was mainly because of the psychological burden, sense of futility of the treatment (46), and high cost of the treatment (47).

Health-seeking in infertility care is seen from a curative aspect rather than a preventive or rehabilitative perspective. Our study also presents the curative aspects of infertility treatment. The curative aspects include giving a wide range of herbal medicines and providing physiotherapeutic and naturopathic care. Similar to other studies (48), the current study shows that herbal medicines are the main course of treatment in the study area. The openness of the problem in the community has made choices and options to manage infertility easily available. The women follow each piece of advice given by a healer. The advice of the *bhagat* includes following certain restrictions, withdrawing from specific food items, and performing rituals related to witchcraft on a full moon or no moon night.

Furthermore, the women consume herbs, receive *Bhutgath* massages, sacrifice animals or birds, and offer alcohol and money as part of the treatment. These practices, that are very common in the study villages, have some side effects. Side effects include nausea, swelling of the eyes, and restlessness. Because of the *dagani* (burn), women experience extreme pain. The constant demand for money and alcohol from the *bhagat* creates many problems.

Primary infertile and young women avail services from more providers and have treatment for a longer duration than sub-fertile women. As the desire to have children is higher among primary infertile couples than secondary, treatment seeking is also high among them (4, 28, 41, 43). The literature suggests that treatment seeking for infertility generally starts within 1–3 years of marriage (4, 21). In our study, half of the primary infertile and sub-fertile women start the treatment after 24 and 36 months of marriage, respectively. Women are the primary care seekers for infertility, and men's involvement in treatment seeking seems to be very limited. A study by Pasch (49) made similar findings, whereby husbands had low infertility treatment-seeking instances, and most of the treatment was done for the wife. It indicates that women feel more pressure from family and community to seek infertility treatment (37). Hence, infertility may be more stressful for women than men (50).

More than half of the primary infertile women were still taking treatment at the time of the survey, and nearly 42% said they had discontinued the treatment and would look for other options. The options included bringing a co-wife, fostering a child, accepting childlessness, or abandoning each other. These coping mechanisms are unique to the tribal communities in the region. A tribal specific study highlighted that coping mechanisms for infertility, such as child adoption, are primarily influenced by perceived causes of infertility. Though this study did not find such association.

## Conclusion

Though the study area is not far from two metropolitan cities, Mumbai and Thane, modernisation, urbanisation, education, and technological advancements have not touched the lives of the people of Jawhar. These villages are not even sufficiently connected with the outer world. The level of education is extremely low, people live below the poverty line, and women's role is measured by procreation. Different myths and cultural malpractices still exist in these rural tribal areas. The community plays a significant role in an individual's life. With supportive attitudes in the community, infertile women suffer less. Lack of resources and constant pressure for childbearing from the community and family push these women to avail services from traditional healers in unsafe and unhygienic conditions. Receiving treatment from formal health care providers, higher literacy, better coping mechanisms, and better support mechanisms have been seen to enable these women to live happily. The local authorities should strive to work towards the socio-economic development of the tribal communities and provide good healthcare services at their doorstep. The infertility problem needs to be understood in the context of poverty, tribal belief, and unequal access to healthcare resources.

### Limitation

There is a recall bias in reporting treatment care. The study is silent on who, between couple, suffers with infertility, however, the treatment is primarily carried out by women. The results of the study can only be generalised in the tribal areas of Maharashtra. The poor reflections regarding the public health facilities, staff and the services are based on the experience and perceptions of the respondents.

### Study contributions

The current study used primary data collected from tribal community setting by face-to-face interviewing. It involves mix method research design obtaining cross-sectional data to answer the research questions on treatment seeking practices. The study demonstrates unique and culture specific issues and practices of tribal population on infertility treatment.

By researchers' understanding, this is one of the few studies conducted on infertility issues in the given study area or tribes that points out various approaches and practices of health care seeking. The study shows how community drives health care practices and person's treatment seeking path.

## Data Availability

The original contributions presented in the study are included in the article, further inquiries can be directed to the corresponding author.

## Footnotes

^1^Primary infertility is the inability to become pregnant or conceive. A woman had primary infertility if, at the time of data collection, she was (1) currently married, aged 15-49 years, staying with her husband for more than two years, (2) had never used any contraception, and (3) never had a pregnancy.

^2^Sub-fertile women included those who had experienced infertility, whether delayed fertility (primary infertile) or secondary infertility. Woman with secondary infertility included (1) currently married women, aged 15-49 years, staying with their husband for more than two years, (2) women who had ever had a conception or live birth, (3) women trying to conceive since their last pregnancy or childbirth and not using any contraceptive or women who reported a delayed conception after previous childbirth for more than two years. In this study, the term applied to women who were experiencing infertility at the time of data collection and had never experienced secondary infertility.

^3^“The shidwa ritual addresses the problem of witchcraft. A paste made of red-coloured powder (Gulal/kumkum) and turmeric is applied to an earthen pot. The pot, a coconut, and a cock are circled seven times over the patient's (the infertile woman) head. The woman sits on the ground with her legs straightened, wrapped in cotton thread. These items are then buried under the earth, and a stone is placed in the pit. The site must never be dug up to prevent the spirit or magic from escaping. Shidwa is done on the river banks under a Sidh tree or on the patient's farm. People from the woman's village are invited to participate. On completion of the ritual, a small feast, where alcohol is served, is arranged for the faith healer and the participants. The faith healer is paid INR 50-100 and gifted a piece of cloth.” (As described by a 46-year-old healthcare worker, educated up to the fourth standard).
